# Acute limb ischemia in young adults secondary to type B aortic dissection: a case report and review of surgical management

**DOI:** 10.1097/RC9.0000000000000941

**Published:** 2026-09-18

**Authors:** Meghdad Ghasemi Gorji, Behdad Ghanami, Yasaman Tavakoli

**Affiliations:** a Department of Vascular Surgery, Shiraz University of Medical Sciences, Shiraz, Iran; b Department of Medicine, Mazandaran University of Medical Sciences, Mazandaran, Sari, Iran

**Keywords:** axillofemoral bypass, case report, femorofemoral bypass, graft infection, lower limb ischemia, type B aortic dissection

## Abstract

**Introduction:**

Type B aortic dissection (TBAD) accounts for approximately 40% of aortic dissection cases and typically arises distal to the left subclavian artery. Its incidence ranges from 0.5 to 6.3 cases per 100 000 person-years, with hospitalizations increasing. TBAD carries a high mortality rate, particularly when complicated by acute limb ischemia. Early diagnosis and multidisciplinary management are critical to improving outcomes.

**Case presentation:**

A 20-year-old Asian male presented to the emergency room with pain and coldness in both lower limbs. CT angiography revealed an acute type B aortic dissection. The patient underwent a right axillofemoral bypass. After 3 weeks, he returned with bleeding due to a graft infection, necessitating graft closure. Subsequently, he developed ischemia again and underwent a left axillofemoral bypass along with a femorofemoral bypass.

**Clinical discussion:**

This case highlights the importance of recognizing atypical presentations of TBAD, particularly when acute bilateral limb ischemia occurs in a young patient. Clinicians should consider aortic pathology even in the absence of classic chest or back pain. Early CT angiography is essential for rapid diagnosis and guiding appropriate management. Individualized revascularization strategies and close postoperative monitoring are critical to address complications and prevent recurrent ischemia.

**Conclusions:**

Early recognition of TBAD is critical when acute limb ischemia occurs, even in young patients without typical symptoms. Prompt imaging and tailored revascularization can be lifesaving and help prevent recurrent ischemic complications.

## Introduction

Aortic dissection (AD) is a life-threatening condition, with type B aortic dissection (TBAD) accounting for about 39.5% of cases^[^1^]^. The incidence of acute TBAD in adults ranges from 0.5 to 6.3 per 100 000 person-years, with hospital admissions rising over time^[^2^]^. TBAD carries high short-term mortality, with a 30-day adjusted hazard ratio of 2.1 compared with the general hypertensive population^[^1^]^. Acute limb ischemia is a rare but fatal complication of TBAD, occurring in 5.7–30.0% of cases^[^3,4^]^. Timely diagnosis and multidisciplinary management are essential to improve outcomes^[^4,5^]^. This report describes a young man who presented with bilateral lower-limb ischemia and was subsequently diagnosed with TBAD. This paper has been reported in line with the SCARE 2025 criteria^[^6^]^.

HIGHLIGHTS

- Acute limb ischemia can be the initial and predominant presentation of type B aortic dissection (TBAD) even in very young adults, highlighting the need to include aortic pathology in the differential diagnosis of bilateral lower-limb ischemia.
- This report describes a 20-year-old patient with TBAD causing bilateral iliac malperfusion due to thrombosis of the false lumen, an uncommon mechanism, particularly in patients without typical risk factors.
- Extra-anatomic revascularization (axillofemoral and femorofemoral bypass) successfully restored limb perfusion when immediate aortic repair was not feasible, demonstrating a practical staged surgical strategy.
- The case illustrates the risk of postoperative complications such as graft infection and thrombosis, requiring repeat revascularization and close postoperative surveillance.

## Case presentation

A 20-year-old Asian man presented to the emergency department with bilateral leg pain and coldness that progressed from tingling to severe agony. Examination showed absent femoral pulses, cold extremities, and a blood pressure of 220/140 mmHg.

CT angiography for a suspected saddle embolism showed an acute TBAD with thrombotic occlusion of a branch of the right renal artery. The superior mesenteric artery, celiac trunk, and right renal artery arose from a narrow true lumen, while both iliac arteries originated from a thrombosed false lumen (Fig. 1). Right axillofemoral bypass restored blood flow and stabilized the patient (Fig. 2). Three weeks after discharge, the patient returned with drainage and bleeding from the right inguinal graft site. Because of suspicion of graft infection or a surgical complication, the graft was closed to control the problem.

**Figure 1.**
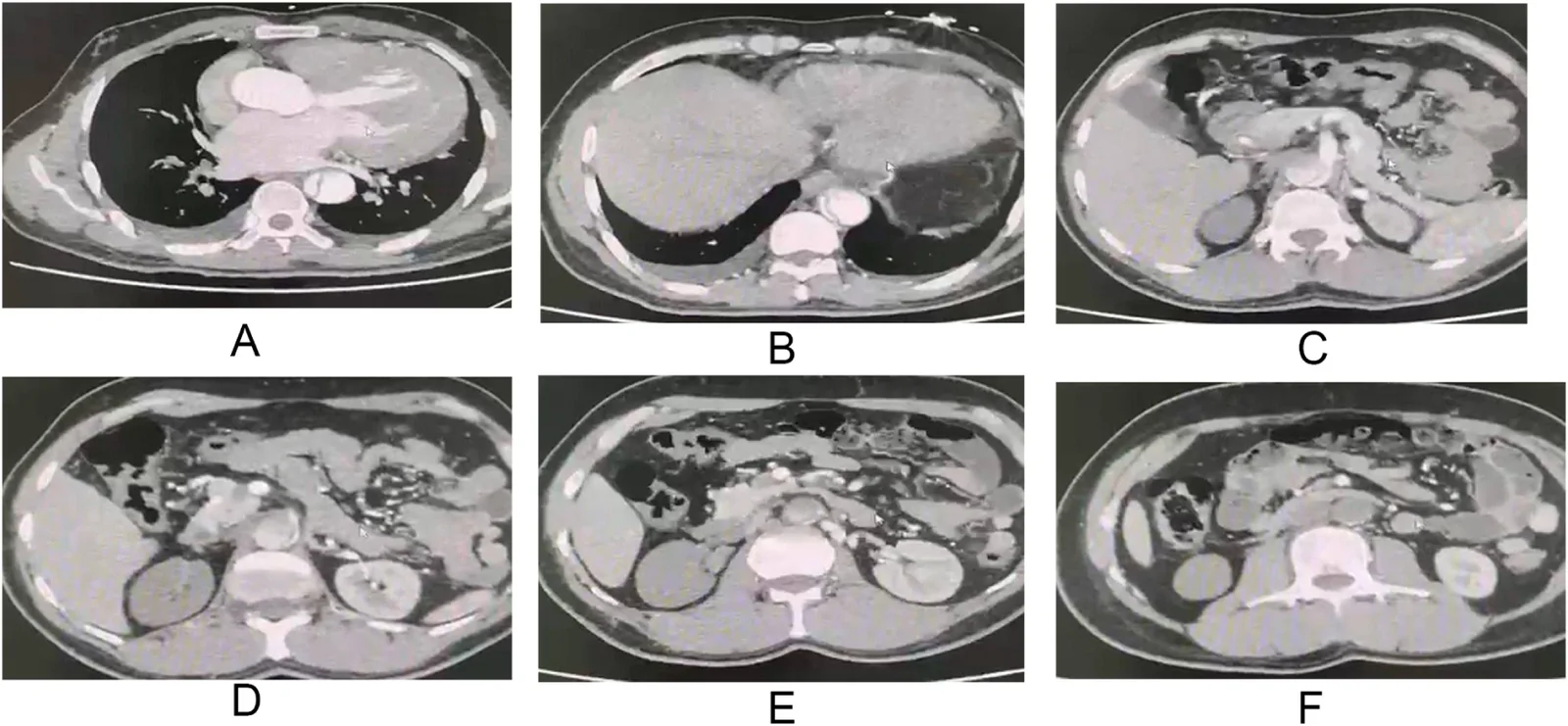
(A) True and false lumens are seen in the descending aorta on a contrast-enhanced spiral chest CT scan. (B) There is significant narrowing of the true lumen in the descending aorta on a contrast-enhanced spiral chest CT scan. (C) There is evidence of partial thrombosis in the true lumen. The right kidney has no vascular supply. (D) The right kidney is necrotic and lacks vascular supply. (E) Extensive necrosis extends to the lower pole of the right kidney. (F) There is complete thrombosis of the aorta from the infrarenal region.

**Figure 2.**
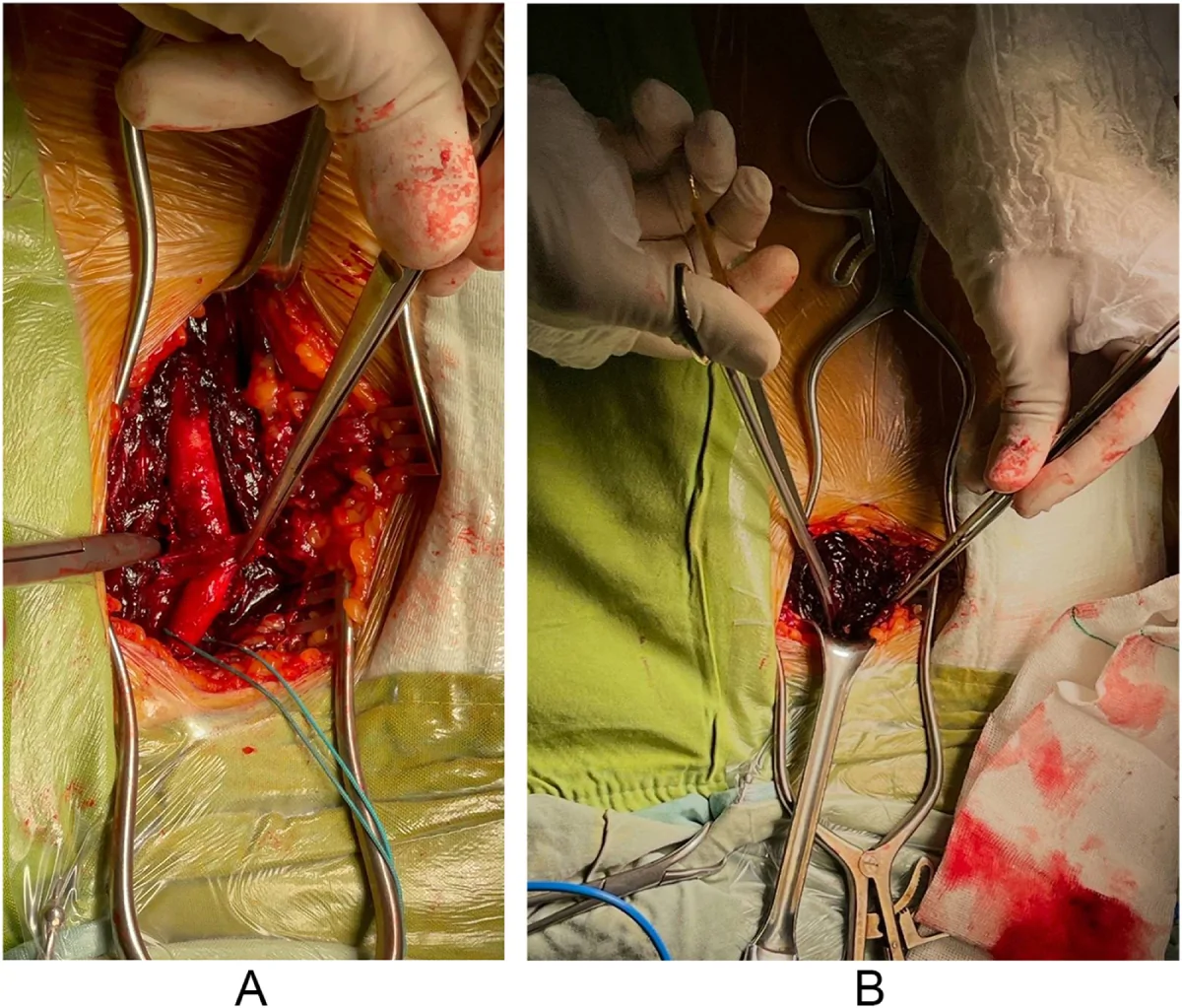
(A) Common femoral artery with dissection-related hematoma. (B) Pre-arterial hematoma secondary to dissection, accompanied by limb ischemia.

Forty-eight hours later, in the intensive care unit, he developed recurrent symptoms of lower-limb ischemia. A repeat CT angiography confirmed thrombosis of the right axillofemoral bypass graft (Fig. 3). He subsequently underwent left axillofemoral bypass with a prosthetic graft and a femorofemoral crossover bypass using the saphenous vein, which reestablished bilateral lower-limb perfusion.

**Figure 3.**
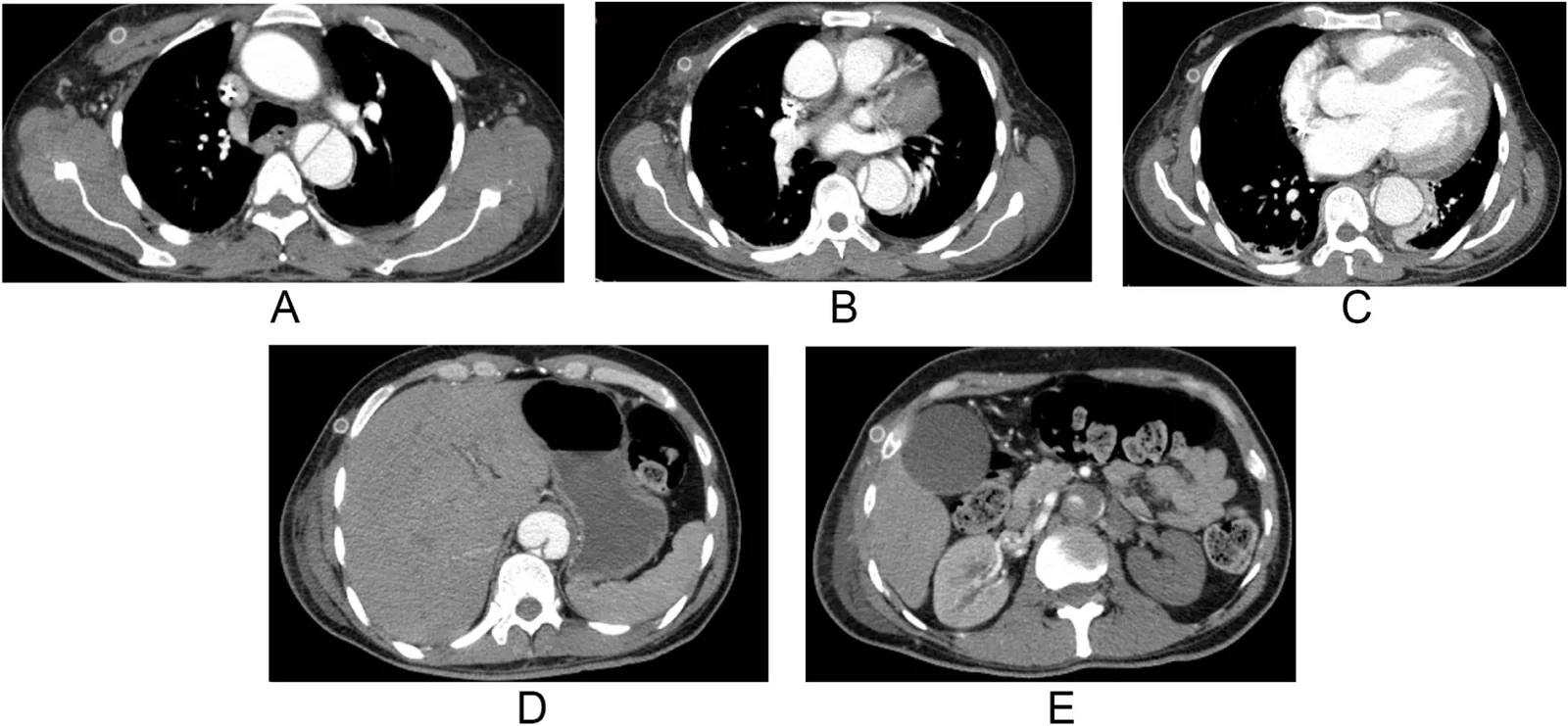
(A) True and false lumens are visible in the descending aorta on a contrast-enhanced spiral chest CT image, with thrombosis present in the right-sided graft. (B) Further inferiorly, the dissection causes significant narrowing of the true lumen. (C) The descending aorta shows significant narrowing of the true lumen, surrounded by a hematoma. Thrombosis is also observed in the right-sided graft. (D) An opening is visible between the true and false lumens, with thrombosis also noted in the right-sided graft. (E) There is complete thrombosis of the aorta, along with thrombosis in the right-sided graft.

## Discussion

Supplemental Digital Content Table 1, available at: https://links.lww.com/IJSCR/A155, demonstrates that TBAD-associated lower-limb ischemia is reported predominantly in older hypertensive males presenting with chest, back, or abdominal pain and signs of limb ischemia. Surgeons primarily use bypass grafts (axillofemoral, femorofemoral) to restore limb perfusion. Endovascular techniques (TEVAR, balloon fenestration) are also frequently employed to balance pressures between the aortic lumens. The outcomes were variable. Many patients survived the initial event and treatment. However, some patients died due to complications such as aortic rupture, multi-organ failure, intracranial hemorrhage, and myocardial infarction. One patient became brain-dead, and another required limb amputation.

TBAD typically occurs in the aorta distal to the left subclavian artery^[^3^]^. Common risk factors include hypertension, older age, and connective tissue disorders; it can also happen after surgery or severe physical or emotional stress^[^7–11^]^. In contrast, our patient was young and healthy, with no identifiable risk factors. TBAD presents variably, most often with acute chest or back pain that radiates posteriorly^[^3^]^. Some patients remain asymptomatic, while others develop abdominal pain, lower-limb ischemia, or organ malperfusion^[^3,10,12^]^.

Initial TBAD management focuses on optimal medical therapy (OMT) to control blood pressure and heart rate, usually with β-blockers^[^13^]^. In complicated TBAD, TEVAR improves aortic remodeling and reduces aorta-specific mortality compared with medical therapy alone^[^14,15^]^. In uncomplicated TBAD, clinicians use OMT as first-line therapy and reserve TEVAR for patients with high-risk anatomical or clinical features^[^16^]^. Open surgery remains the preferred treatment for patients with connective tissue disorders such as Marfan syndrome or Loeys–Dietz syndrome and for selected cases where TEVAR is unsuitable^[^17^]^. Hybrid approaches that combine surgical and endovascular techniques effectively treat extensive aortic disease and are used in patients with connective tissue disorders^[^18^]^. Although OMT and TEVAR form the foundation of TBAD management, malperfusion syndromes shift priorities and often require additional intervention^[^19^]^.

Lower limb malperfusion is a life-threatening complication of TBAD that occurs in many complicated cases^[^12^]^. It results from branch-artery occlusion or thrombosis, or from true lumen compression by the dissection flap or an expanding false lumen^[^20^]^. Lower limb malperfusion presents as acute limb ischemia with pain, pallor, paresthesia, paralysis, and absent pulses^[^21^]^. It often coexists with malperfusion in other territories, including the renal (40%) and visceral (25%) circulations^[^12^]^. Distinguishing lower limb malperfusion from peripheral embolism is critical, especially when limb ischemia occurs without typical thoracoabdominal symptoms. In this case, the patient presented with sudden-onset lower-limb ischemia without chest or back pain, which complicated the diagnosis.

Treating TBAD with lower limb ischemia remains challenging and requires patient-specific decisions. Because this complication is rare, high-quality evidence is limited^[^3,11^]^. Treatment aims to rapidly restore limb perfusion while addressing the AD and any associated aneurysm^[^11^]^. Clinicians should base treatment decisions on the patient’s condition, the cause of ischemia, dissection anatomy, and local expertise^[^9^]^.

TEVAR restores limb perfusion in many patients with TBAD-related lower-limb ischemia by sealing the entry tear, reducing false lumen pressure, and re-expanding the true lumen, but if ischemia persists, additional revascularization procedures will be necessary^[^3,22^]^.

Endovascular fenestration offers another option, particularly for dynamic malperfusion, by creating a channel between the true and false lumens, equalizing pressure, and improving distal flow. However, fenestration alone may not resolve ischemia and often requires stenting or additional interventions^[^19,23^]^.

In patients with severe limb ischemia, or when immediate aortic repair is not feasible, extra-anatomic bypass procedures (axillofemoral, axillobifemoral, or femorofemoral) rapidly restore perfusion. These procedures are particularly useful in bilateral lower-limb ischemia. However, these procedures do not correct the dissection and require ongoing surveillance and, often, later aortic repair^[^12,24^]^.

Other treatment options include iliac artery stenting when iliac obstruction causes malperfusion, and the Provisional Extension To Induce Complete Attachment technique, which combines proximal stent grafting with distal bare-metal stenting to expand the true lumen and promote aortic remodeling^[^25,26^]^. Open aortic repair remains a last-line option because it carries higher morbidity and mortality than endovascular approaches^[^10^]^.

In our case, the patient presented with bilateral lower-extremity ischemia caused by complete obstruction of both iliac arteries secondary to TBAD. Although TEVAR is the standard treatment for complicated TBAD, in this patient, it was not expected to provide sufficiently rapid restoration of lower-extremity perfusion because of severe bilateral iliac malperfusion and complete distal outflow obstruction. Therefore, we performed a right axillofemoral bypass as a fast, feasible revascularization strategy. When the graft later failed due to thrombosis and suspected infection, a left axillofemoral bypass and a saphenous-vein femorofemoral bypass were performed to reestablish and maintain bilateral blood flow. In addition to surgical treatment, we also managed blood pressure with labetalol and systemic anticoagulation with heparin during hospitalization and apixaban after discharge to reduce the risk of graft thrombosis and recurrent ischemia. The patient remained stable with adequate limb perfusion and no ongoing malperfusion after the second surgery. Therefore, we initiated regular clinical surveillance after discharge and will perform definitive aortic repair if aneurysmal progression, recurrent malperfusion, or other complications occur.

The present case is notable for several reasons. First, TBAD occurred in a 20-year-old patient without recognized predisposing factors. Second, bilateral acute limb ischemia was the sole presenting manifestation in the absence of chest, back, or abdominal pain. Third, both iliac arteries originated from a completely thrombosed false lumen, producing severe bilateral malperfusion. Finally, successful limb salvage required staged extra-anatomic revascularization following graft-related complications, highlighting the complexity of management in this uncommon presentation.

## Conclusion

This case of a young man with TBAD and bilateral lower-limb ischemia highlights the variable presentation of this condition and the need for timely, accurate diagnosis, even without classic chest or back pain. The graft infection and recurrent ischemia that required multiple revascularization procedures illustrate the complexity of this condition and the risk of morbidity, despite initially successful revascularization. This case demonstrates the value of multidisciplinary care and close follow-up for these patients.

## Data Availability

All relevant data are included within the article. Further details can be made available from the corresponding author upon reasonable request.
